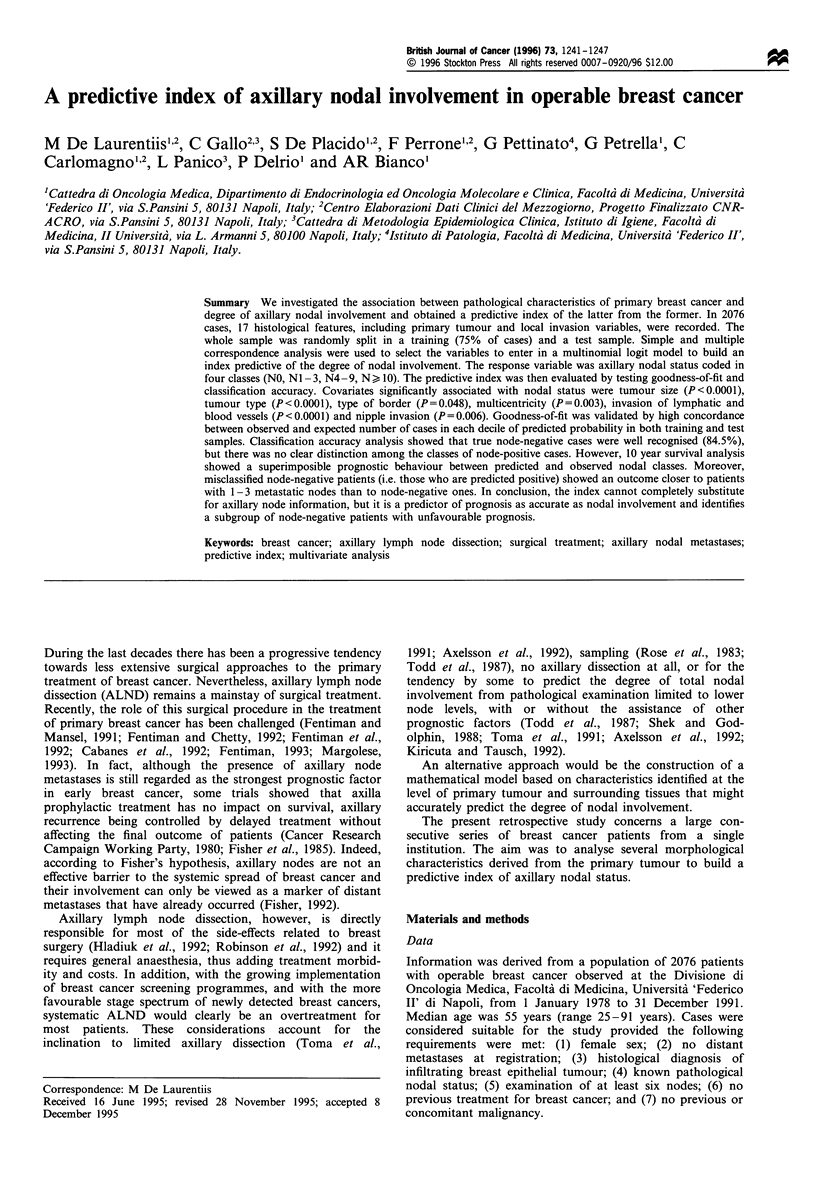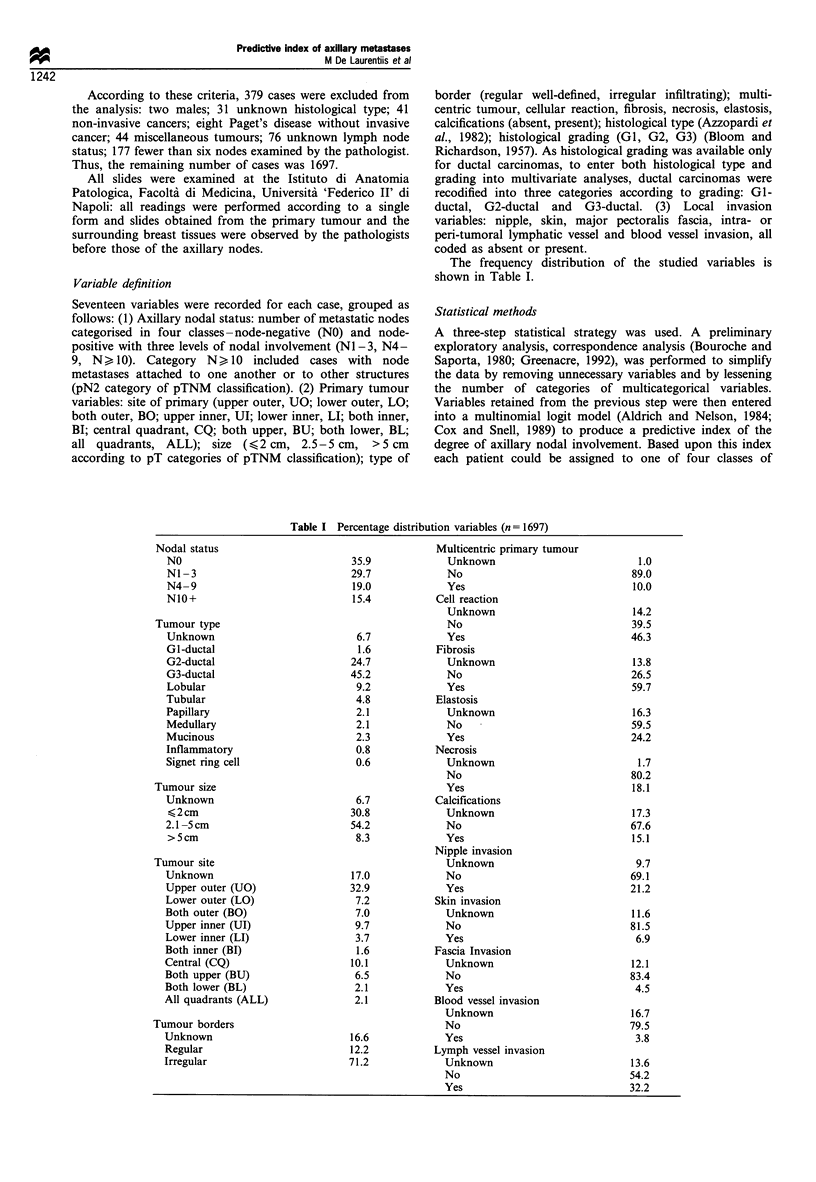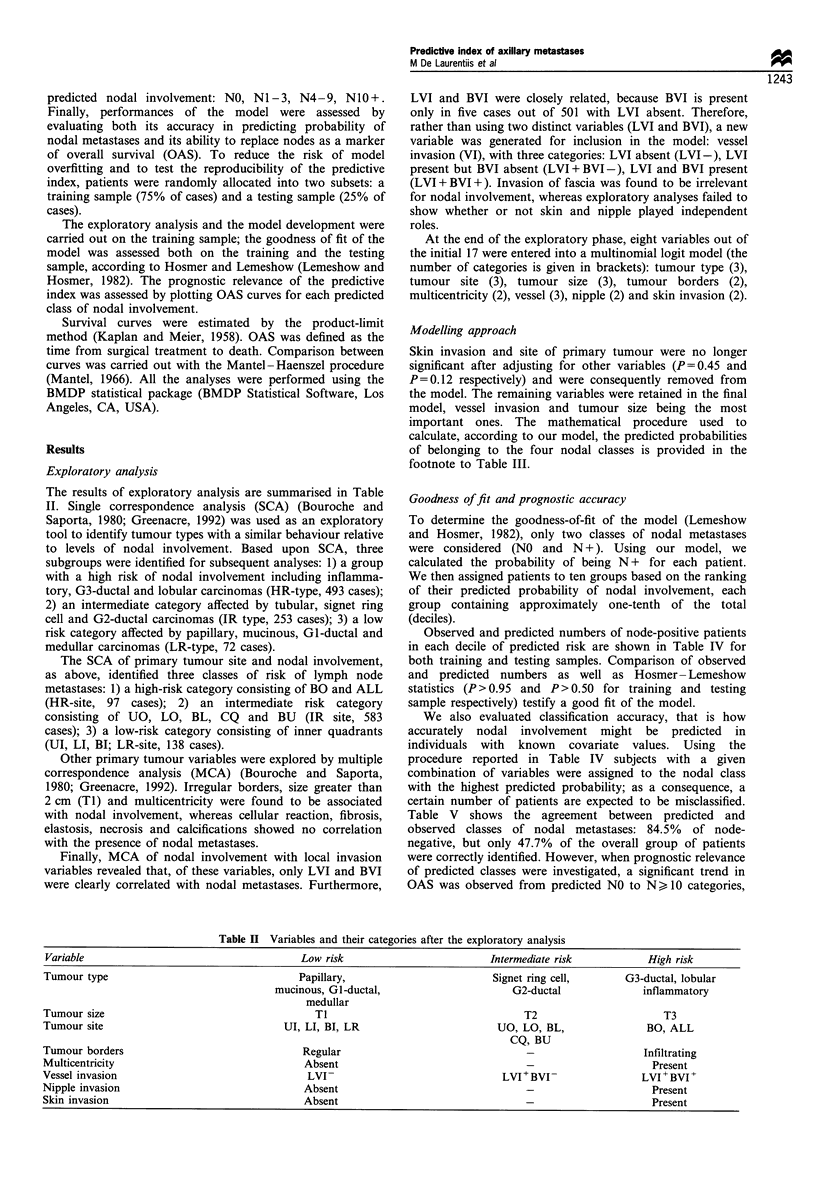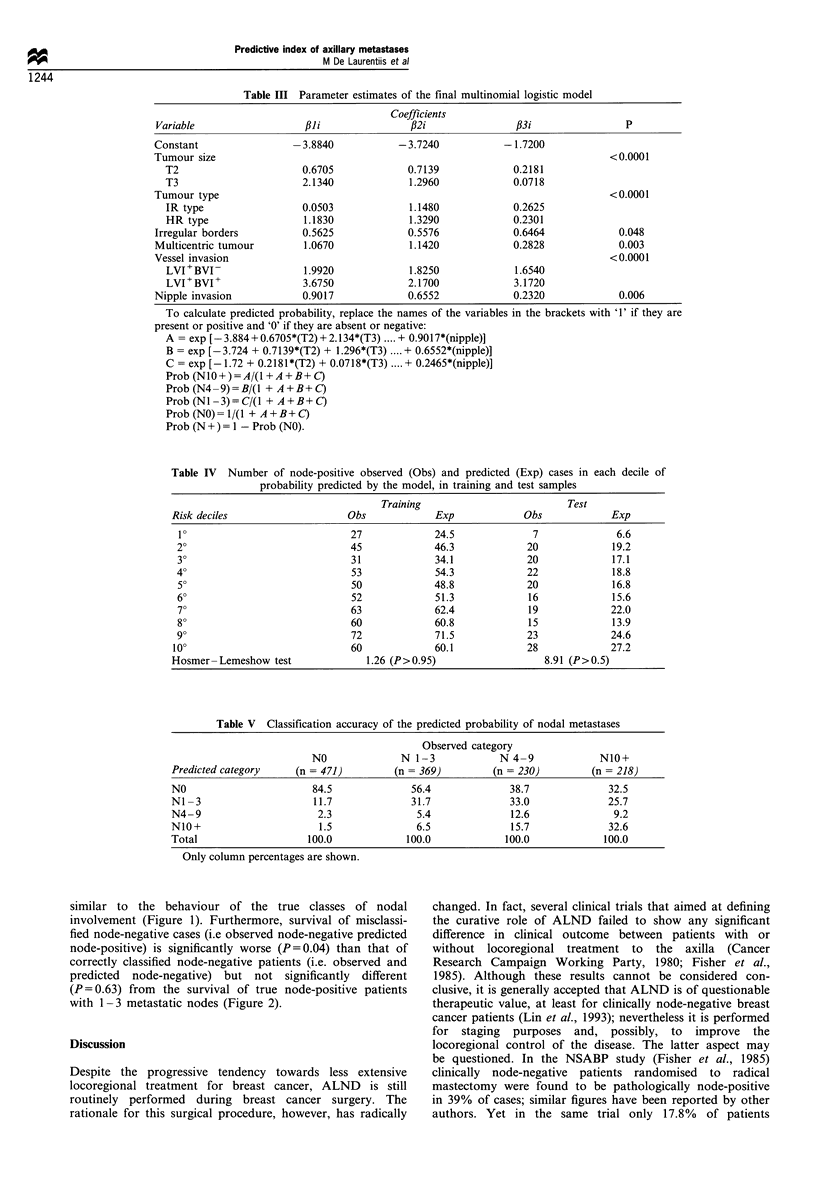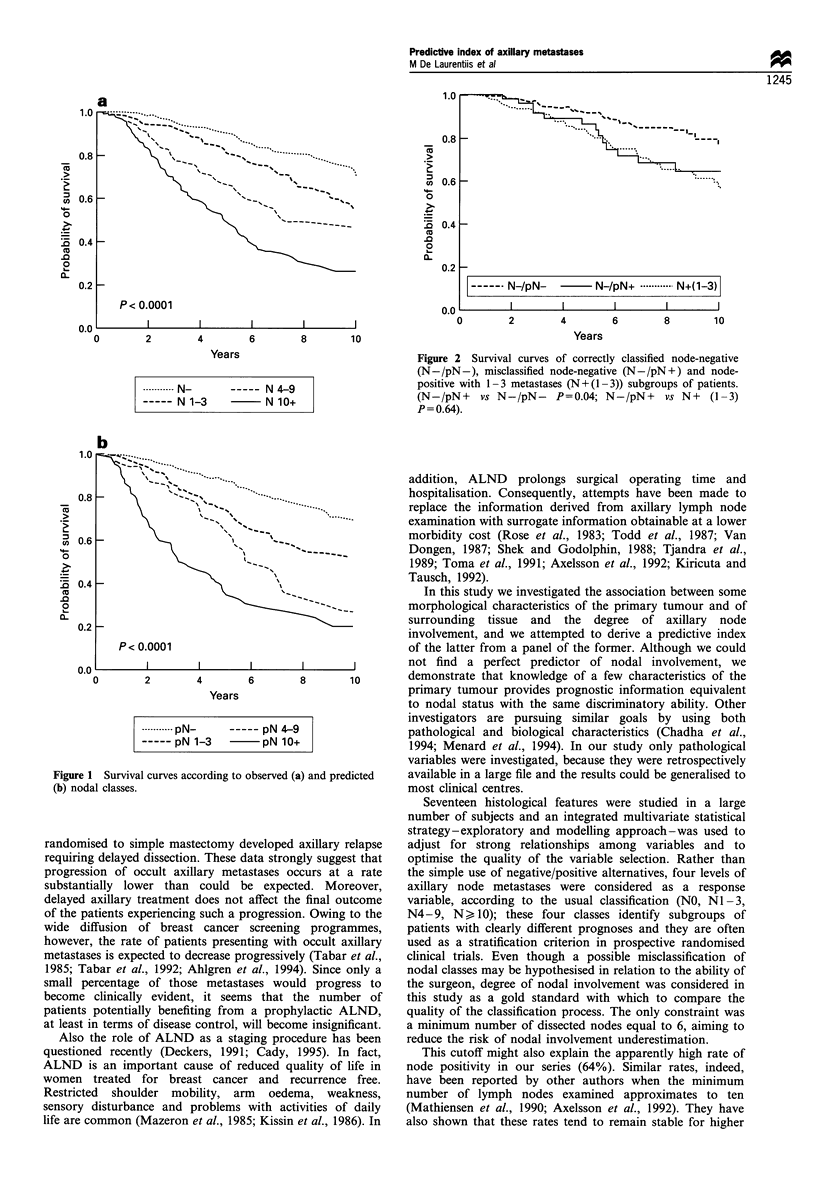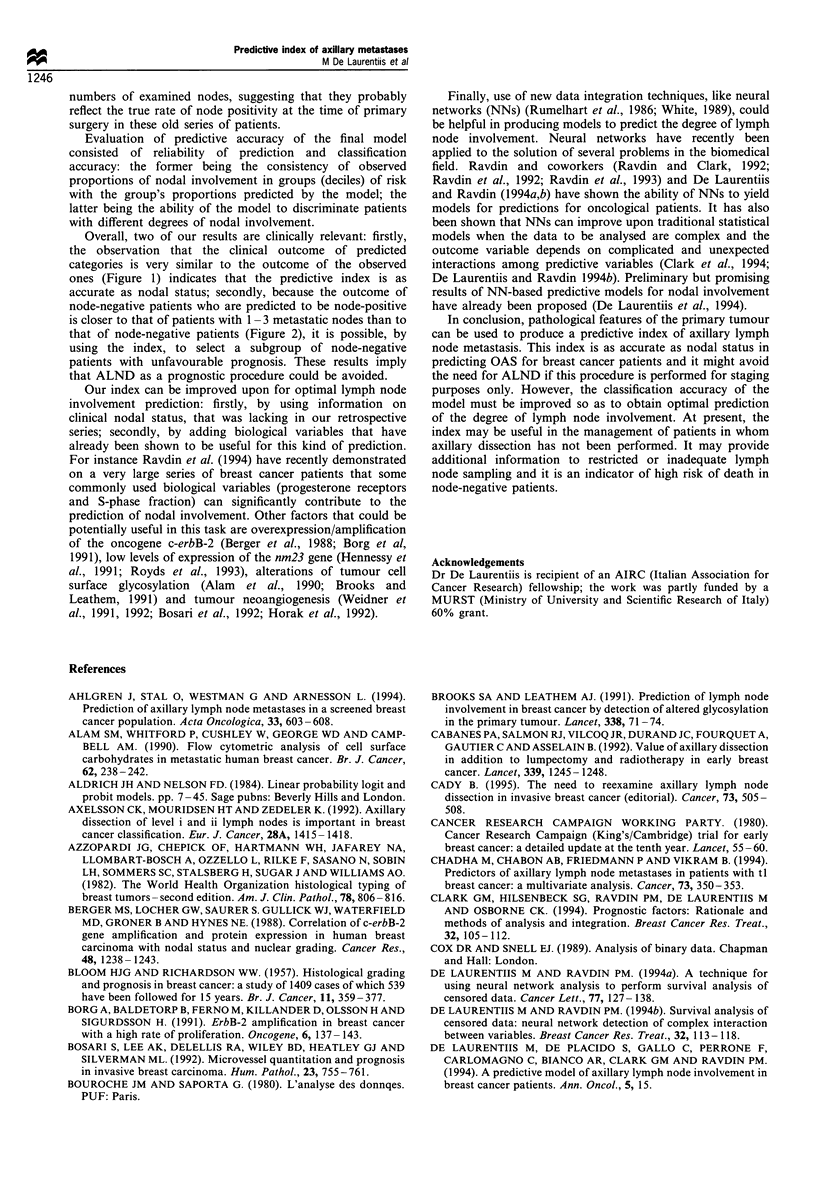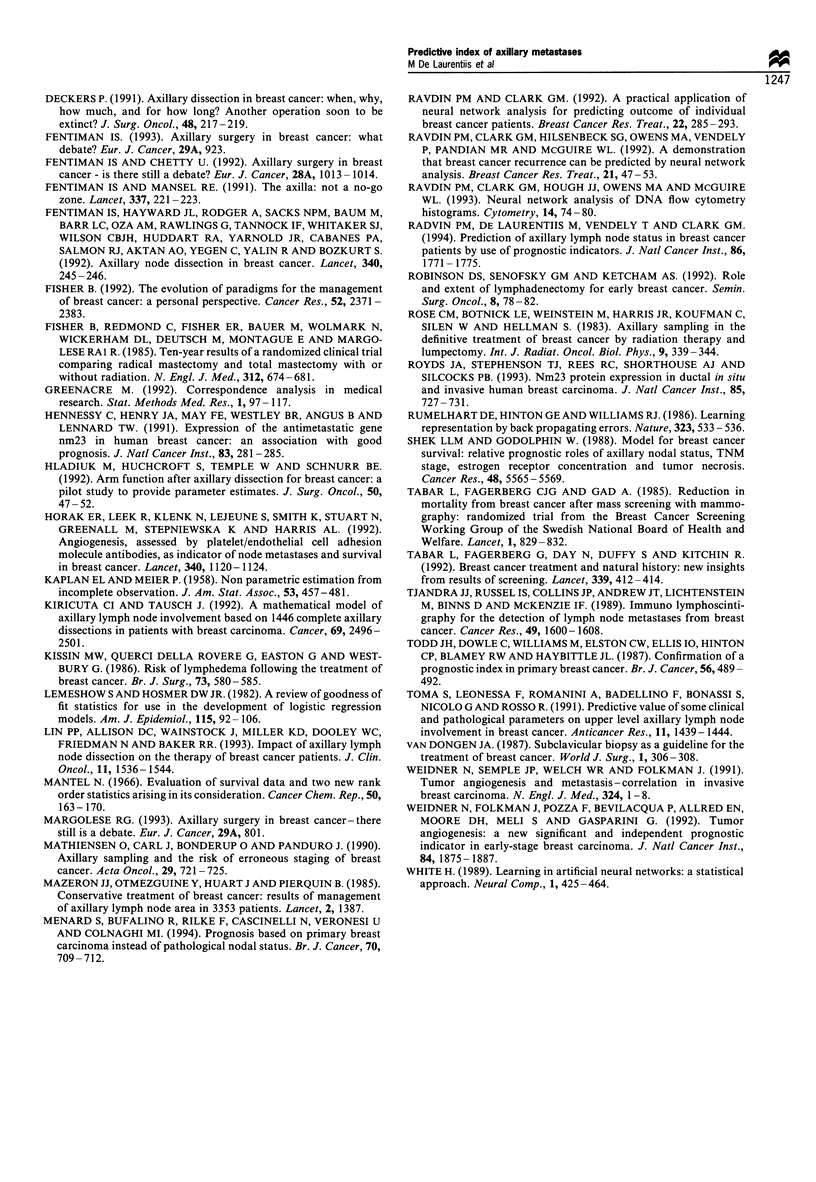# A predictive index of axillary nodal involvement in operable breast cancer.

**DOI:** 10.1038/bjc.1996.238

**Published:** 1996-05

**Authors:** M. De Laurentiis, C. Gallo, S. De Placido, F. Perrone, G. Pettinato, G. Petrella, C. Carlomagno, L. Panico, P. Delrio, A. R. Bianco

**Affiliations:** Dipartimento di Endocrinologia ed Oncologia Molecolare e Clinica, Facoltà di Medicina, Università Feferico II, Napoli, Italy.

## Abstract

We investigated the association between pathological characteristics of primary breast cancer and degree of axillary nodal involvement and obtained a predictive index of the latter from the former. In 2076 cases, 17 histological features, including primary tumour and local invasion variables, were recorded. The whole sample was randomly split in a training (75% of cases) and a test sample. Simple and multiple correspondence analysis were used to select the variables to enter in a multinomial logit model to build an index predictive of the degree of nodal involvement. The response variable was axillary nodal status coded in four classes (N0, N1-3, N4-9, N > or = 10). The predictive index was then evaluated by testing goodness-of-fit and classification accuracy. Covariates significantly associated with nodal status were tumour size (P < 0.0001), tumour type (P < 0.0001), type of border (P = 0.048), multicentricity (P = 0.003), invasion of lymphatic and blood vessels (P < 0.0001) and nipple invasion (P = 0.006). Goodness-of-fit was validated by high concordance between observed and expected number of cases in each decile of predicted probability in both training and test samples. Classification accuracy analysis showed that true node-positive cases were well recognised (84.5%), but there was no clear distinction among the classes of node-positive cases. However, 10 year survival analysis showed a superimposible prognostic behaviour between predicted and observed nodal classes. Moreover, misclassified node-negative patients (i.e. those who are predicted positive) showed an outcome closer to patients with 1-3 metastatic nodes than to node-negative ones. In conclusion, the index cannot completely substitute for axillary node information, but it is a predictor of prognosis as accurate as nodal involvement and identifies a subgroup of node-negative patients with unfavourable prognosis.


British Journal of Cancer (1996) 73, 1241-1247
© 1996 Stockton Press All rights reserved 0007-0920/96 $12.00

### A predictive index of axillary nodal involvement in operable breast cancer

M De Laurentiis[1,2], C Gallo[2,3], S De Placido[1,2], F Perrone[1,2], G Pettinato[4], G Petrella[1], C Carlomagno[1,2], L Panico[3], P Delrio[1] and AR Bianco[1]

[1]*Cattedra di Oncologia Medica, Dipartimento di Endocrinologia ed Oncologia Molecolare e Clinica, Facoltà di Medicina, Università 'Federico II', via S.Pansini 5, 80131 Napoli, Italy;* [2]*Centro Elaborazioni Dati Clinici del Mezzogiorno, Progetto Finalizzato CNR-ACRO, via S.Pansini 5, 80131 Napoli, Italy;* [3]*Cattedra di Metodologia Epidemiologica Clinica, Istituto di Igiene, Facoltà di Medicina, II Università, via L. Armanni 5, 80100 Napoli, Italy;* [4]*Istituto di Patologia, Facoltà di Medicina, Università 'Federico II', via S.Pansini 5, 80131 Napoli, Italy.*

**Summary** We investigated the association between pathological characteristics of primary breast cancer and degree of axillary nodal involvement and obtained a predictive index of the latter from the former. In 2076 cases, 17 histological features, including primary tumour and local invasion variables, were recorded. The whole sample was randomly split in a training (75% of cases) and a test sample. Simple and multiple correspondence analysis were used to select the variables to enter in a multinomial logit model to build an index predictive of the degree of nodal involvement. The response variable was axillary nodal status coded in four classes (N0, N1-3, N4-9, N≥10). The predictive index was then evaluated by testing goodness-of-fit and classification accuracy. Covariates significantly associated with nodal status were tumour size ($P<0.0001$), tumour type ($P<0.0001$), type of border ($P=0.048$), multicentricity ($P=0.003$), invasion of lymphatic and blood vessels ($P<0.0001$) and nipple invasion ($P=0.006$). Goodness-of-fit was validated by high concordance between observed and expected number of cases in each decile of predicted probability in both training and test samples. Classification accuracy analysis showed that true node-negative cases were well recognised (84.5%), but there was no clear distinction among the classes of node-positive cases. However, 10 year survival analysis showed a superimposible prognostic behaviour between predicted and observed nodal classes. Moreover, misclassified node-negative patients (i.e. those who are predicted positive) showed an outcome closer to patients with 1-3 metastatic nodes than to node-negative ones. In conclusion, the index cannot completely substitute for axillary node information, but it is a predictor of prognosis as accurate as nodal involvement and identifies a subgroup of node-negative patients with unfavourable prognosis.

**Keywords:** breast cancer; axillary lymph node dissection; surgical treatment; axillary nodal metastases; predictive index; multivariate analysis

During the last decades there has been a progressive tendency towards less extensive surgical approaches to the primary treatment of breast cancer. Nevertheless, axillary lymph node dissection (ALND) remains a mainstay of surgical treatment. Recently, the role of this surgical procedure in the treatment of primary breast cancer has been challenged (Fentiman and Mansel, 1991; Fentiman and Chetty, 1992; Fentiman *et al.*, 1992; Cabanes *et al.*, 1992; Fentiman, 1993; Margolese, 1993). In fact, although the presence of axillary node metastases is still regarded as the strongest prognostic factor in early breast cancer, some trials showed that axilla prophylactic treatment has no impact on survival, axillary recurrence being controlled by delayed treatment without affecting the final outcome of patients (Cancer Research Campaign Working Party, 1980; Fisher *et al.*, 1985). Indeed, according to Fisher's hypothesis, axillary nodes are not an effective barrier to the systemic spread of breast cancer and their involvement can only be viewed as a marker of distant metastases that have already occurred (Fisher, 1992).

Axillary lymph node dissection, however, is directly responsible for most of the side-effects related to breast surgery (Hladiuk *et al.*, 1992; Robinson *et al.*, 1992) and it requires general anaesthesia, thus adding treatment morbidity and costs. In addition, with the growing implementation of breast cancer screening programmes, and with the more favourable stage spectrum of newly detected breast cancers, systematic ALND would clearly be an overtreatment for most patients. These considerations account for the inclination to limited axillary dissection (Toma *et al.*, 1991; Axelsson *et al.*, 1992), sampling (Rose *et al.*, 1983; Todd *et al.*, 1987), no axillary dissection at all, or for the tendency by some to predict the degree of total nodal involvement from pathological examination limited to lower node levels, with or without the assistance of other prognostic factors (Todd *et al.*, 1987; Shek and Godolphin, 1988; Toma *et al.*, 1991; Axelsson *et al.*, 1992; Kiricuta and Tausch, 1992).

An alternative approach would be the construction of a mathematical model based on characteristics identified at the level of primary tumour and surrounding tissues that might accurately predict the degree of nodal involvement.

The present retrospective study concerns a large consecutive series of breast cancer patients from a single institution. The aim was to analyse several morphological characteristics derived from the primary tumour to build a predictive index of axillary nodal status.

### Materials and methods

### *Data*

Information was derived from a population of 2076 patients with operable breast cancer observed at the Divisione di Oncologia Medica, Facoltà di Medicina, Università 'Federico II' di Napoli, from 1 January 1978 to 31 December 1991. Median age was 55 years (range 25-91 years). Cases were considered suitable for the study provided the following requirements were met: (1) female sex; (2) no distant metastases at registration; (3) histological diagnosis of infiltrating breast epithelial tumour; (4) known pathological nodal status; (5) examination of at least six nodes; (6) no previous treatment for breast cancer; and (7) no previous or concomitant malignancy.

Correspondence: M De Laurentiis
Received 16 June 1995; revised 28 November 1995; accepted 8 December 1995

According to these criteria, 379 cases were excluded from the analysis: two males; 31 unknown histological type; 41 non-invasive cancers; eight Paget's disease without invasive cancer; 44 miscellaneous tumours; 76 unknown lymph node status; 177 fewer than six nodes examined by the pathologist. Thus, the remaining number of cases was 1697.

All slides were examined at the Istituto di Anatomia Patologica, Facoltà di Medicina, Università 'Federico II' di Napoli: all readings were performed according to a single form and slides obtained from the primary tumour and the surrounding breast tissues were observed by the pathologists before those of the axillary nodes.

### *Variable definition*

Seventeen variables were recorded for each case, grouped as follows: (1) Axillary nodal status: number of metastatic nodes categorised in four classes – node-negative (N0) and node-positive with three levels of nodal involvement (N1 – 3, N4 – 9, N⩾10). Category N⩾10 included cases with node metastases attached to one another or to other structures (pN2 category of pTNM classification). (2) Primary tumour variables: site of primary (upper outer, UO; lower outer, LO; both outer, BO; upper inner, UI; lower inner, LI; both inner, BI; central quadrant, CQ; both upper, BU; both lower, BL; all quadrants, ALL); size (⩽2 cm, 2.5 – 5 cm, >5 cm according to pT categories of pTNM classification); type of border (regular well-defined, irregular infiltrating); multicentric tumour, cellular reaction, fibrosis, necrosis, elastosis, calcifications (absent, present); histological type (Azzopardi *et al*., 1982); histological grading (G1, G2, G3) (Bloom and Richardson, 1957). As histological grading was available only for ductal carcinomas, to enter both histological type and grading into multivariate analyses, ductal carcinomas were recodified into three categories according to grading: G1-ductal, G2-ductal and G3-ductal. (3) Local invasion variables: nipple, skin, major pectoralis fascia, intra- or peri-tumoral lymphatic vessel and blood vessel invasion, all coded as absent or present.

The frequency distribution of the studied variables is shown in Table I.

### *Statistical methods*

A three-step statistical strategy was used. A preliminary exploratory analysis, correspondence analysis (Bouroche and Saporta, 1980; Greenacre, 1992), was performed to simplify the data by removing unnecessary variables and by lessening the number of categories of multicategorical variables. Variables retained from the previous step were then entered into a multinomial logit model (Aldrich and Nelson, 1984; Cox and Snell, 1989) to produce a predictive index of the degree of axillary nodal involvement. Based upon this index each patient could be assigned to one of four classes of

**Table I** Percentage distribution variables ($n = 1697$)

| | | | |
|---|---|---|---|
| Nodal status | | Multicentric primary tumour | |
| N0 | 35.9 | Unknown | 1.0 |
| N1 – 3 | 29.7 | No | 89.0 |
| N4 – 9 | 19.0 | Yes | 10.0 |
| N10+ | 15.4 | Cell reaction | |
| | | Unknown | 14.2 |
| Tumour type | | No | 39.5 |
| Unknown | 6.7 | Yes | 46.3 |
| G1-ductal | 1.6 | Fibrosis | |
| G2-ductal | 24.7 | Unknown | 13.8 |
| G3-ductal | 45.2 | No | 26.5 |
| Lobular | 9.2 | Yes | 59.7 |
| Tubular | 4.8 | Elastosis | |
| Papillary | 2.1 | Unknown | 16.3 |
| Medullary | 2.1 | No | 59.5 |
| Mucinous | 2.3 | Yes | 24.2 |
| Inflammatory | 0.8 | Necrosis | |
| Signet ring cell | 0.6 | Unknown | 1.7 |
| | | No | 80.2 |
| Tumour size | | Yes | 18.1 |
| Unknown | 6.7 | Calcifications | |
| ⩽2 cm | 30.8 | Unknown | 17.3 |
| 2.1 – 5 cm | 54.2 | No | 67.6 |
| >5 cm | 8.3 | Yes | 15.1 |
| | | Nipple invasion | |
| Tumour site | | Unknown | 9.7 |
| Unknown | 17.0 | No | 69.1 |
| Upper outer (UO) | 32.9 | Yes | 21.2 |
| Lower outer (LO) | 7.2 | Skin invasion | |
| Both outer (BO) | 7.0 | Unknown | 11.6 |
| Upper inner (UI) | 9.7 | No | 81.5 |
| Lower inner (LI) | 3.7 | Yes | 6.9 |
| Both inner (BI) | 1.6 | Fascia Invasion | |
| Central (CQ) | 10.1 | Unknown | 12.1 |
| Both upper (BU) | 6.5 | No | 83.4 |
| Both lower (BL) | 2.1 | Yes | 4.5 |
| All quadrants (ALL) | 2.1 | Blood vessel invasion | |
| | | Unknown | 16.7 |
| Tumour borders | | No | 79.5 |
| Unknown | 16.6 | Yes | 3.8 |
| Regular | 12.2 | Lymph vessel invasion | |
| Irregular | 71.2 | Unknown | 13.6 |
| | | No | 54.2 |
| | | Yes | 32.2 |

predicted nodal involvement: N0, N1-3, N4-9, N10+. Finally, performances of the model were assessed by evaluating both its accuracy in predicting probability of nodal metastases and its ability to replace nodes as a marker of overall survival (OAS). To reduce the risk of model overfitting and to test the reproducibility of the predictive index, patients were randomly allocated into two subsets: a training sample (75% of cases) and a testing sample (25% of cases).

The exploratory analysis and the model development were carried out on the training sample; the goodness of fit of the model was assessed both on the training and the testing sample, according to Hosmer and Lemeshow (Lemeshow and Hosmer, 1982). The prognostic relevance of the predictive index was assessed by plotting OAS curves for each predicted class of nodal involvement.

Survival curves were estimated by the product-limit method (Kaplan and Meier, 1958). OAS was defined as the time from surgical treatment to death. Comparison between curves was carried out with the Mantel-Haenszel procedure (Mantel, 1966). All the analyses were performed using the BMDP statistical package (BMDP Statistical Software, Los Angeles, CA, USA).

### Results

### *Exploratory analysis*

The results of exploratory analysis are summarised in Table II. Single correspondence analysis (SCA) (Bouroche and Saporta, 1980; Greenacre, 1992) was used as an exploratory tool to identify tumour types with a similar behaviour relative to levels of nodal involvement. Based upon SCA, three subgroups were identified for subsequent analyses: 1) a group with a high risk of nodal involvement including inflammatory, G3-ductal and lobular carcinomas (HR-type, 493 cases); 2) an intermediate category affected by tubular, signet ring cell and G2-ductal carcinomas (IR type, 253 cases); 3) a low risk category affected by papillary, mucinous, G1-ductal and medullar carcinomas (LR-type, 72 cases).

The SCA of primary tumour site and nodal involvement, as above, identified three classes of risk of lymph node metastases: 1) a high-risk category consisting of BO and ALL (HR-site, 97 cases); 2) an intermediate risk category consisting of UO, LO, BL, CQ and BU (IR site, 583 cases); 3) a low-risk category consisting of inner quadrants (UI, LI, BI; LR-site, 138 cases).

Other primary tumour variables were explored by multiple correspondence analysis (MCA) (Bouroche and Saporta, 1980; Greenacre, 1992). Irregular borders, size greater than 2 cm (T1) and multicentricity were found to be associated with nodal involvement, whereas cellular reaction, fibrosis, elastosis, necrosis and calcifications showed no correlation with the presence of nodal metastases.

Finally, MCA of nodal involvement with local invasion variables revealed that, of these variables, only LVI and BVI were clearly correlated with nodal metastases. Furthermore, LVI and BVI were closely related, because BVI is present only in five cases out of 501 with LVI absent. Therefore, rather than using two distinct variables (LVI and BVI), a new variable was generated for inclusion in the model: vessel invasion (VI), with three categories: LVI absent (LVI−), LVI present but BVI absent (LVI+BVI−), LVI and BVI present (LVI+BVI+). Invasion of fascia was found to be irrelevant for nodal involvement, whereas exploratory analyses failed to show whether or not skin and nipple played independent roles.

At the end of the exploratory phase, eight variables out of the initial 17 were entered into a multinomial logit model (the number of categories is given in brackets): tumour type (3), tumour site (3), tumour size (3), tumour borders (2), multicentricity (2), vessel (3), nipple (2) and skin invasion (2).

### *Modelling approach*

Skin invasion and site of primary tumour were no longer significant after adjusting for other variables ($P=0.45$ and $P=0.12$ respectively) and were consequently removed from the model. The remaining variables were retained in the final model, vessel invasion and tumour size being the most important ones. The mathematical procedure used to calculate, according to our model, the predicted probabilities of belonging to the four nodal classes is provided in the footnote to Table III.

### *Goodness of fit and prognostic accuracy*

To determine the goodness-of-fit of the model (Lemeshow and Hosmer, 1982), only two classes of nodal metastases were considered (N0 and N+). Using our model, we calculated the probability of being N+ for each patient. We then assigned patients to ten groups based on the ranking of their predicted probability of nodal involvement, each group containing approximately one-tenth of the total (deciles).

Observed and predicted numbers of node-positive patients in each decile of predicted risk are shown in Table IV for both training and testing samples. Comparison of observed and predicted numbers as well as Hosmer-Lemeshow statistics ($P>0.95$ and $P>0.50$ for training and testing sample respectively) testify a good fit of the model.

We also evaluated classification accuracy, that is how accurately nodal involvement might be predicted in individuals with known covariate values. Using the procedure reported in Table IV subjects with a given combination of variables were assigned to the nodal class with the highest predicted probability; as a consequence, a certain number of patients are expected to be misclassified. Table V shows the agreement between predicted and observed classes of nodal metastases: 84.5% of node-negative, but only 47.7% of the overall group of patients were correctly identified. However, when prognostic relevance of predicted classes were investigated, a significant trend in OAS was observed from predicted N0 to $N\geqslant10$ categories,

**Table II** Variables and their categories after the exploratory analysis

| *Variable* | *Low risk* | *Intermediate risk* | *High risk* |
|---|---|---|---|
| Tumour type | Papillary, mucinous, G1-ductal, medullar | Signet ring cell, G2-ductal | G3-ductal, lobular inflammatory |
| Tumour size | T1 | T2 | T3 |
| Tumour site | UI, LI, BI, LR | UO, LO, BL, CQ, BU | BO, ALL |
| Tumour borders | Regular | – | Infiltrating |
| Multicentricity | Absent | – | Present |
| Vessel invasion | $LVI^-$ | $LVI^+BVI^-$ | $LVI^+BVI^+$ |
| Nipple invasion | Absent | – | Present |
| Skin invasion | Absent | – | Present |

**Table III** Parameter estimates of the final multinomial logistic model

| *Variable* | *Coefficients* $\beta 1i$ | $\beta 2i$ | $\beta 3i$ | P |
|---|---|---|---|---|
| Constant | −3.8840 | −3.7240 | −1.7200 | |
| Tumour size | | | | <0.0001 |
| T2 | 0.6705 | 0.7139 | 0.2181 | |
| T3 | 2.1340 | 1.2960 | 0.0718 | |
| Tumour type | | | | <0.0001 |
| IR type | 0.0503 | 1.1480 | 0.2625 | |
| HR type | 1.1830 | 1.3290 | 0.2301 | |
| Irregular borders | 0.5625 | 0.5576 | 0.6464 | 0.048 |
| Multicentric tumour | 1.0670 | 1.1420 | 0.2828 | 0.003 |
| Vessel invasion | | | | <0.0001 |
| $LVI^+BVI^-$ | 1.9920 | 1.8250 | 1.6540 | |
| $LVI^+BVI^+$ | 3.6750 | 2.1700 | 3.1720 | |
| Nipple invasion | 0.9017 | 0.6552 | 0.2320 | 0.006 |

To calculate predicted probability, replace the names of the variables in the brackets with '1' if they are present or positive and '0' if they are absent or negative:
A = exp [−3.884 + 0.6705*(T2) + 2.134*(T3) .... + 0.9017*(nipple)]
B = exp [−3.724 + 0.7139*(T2) + 1.296*(T3) .... + 0.6552*(nipple)]
C = exp [−1.72 + 0.2181*(T2) + 0.0718*(T3) .... + 0.2465*(nipple)]
Prob (N10+) = $A/(1+A+B+C)$
Prob (N4−9) = $B/(1+A+B+C)$
Prob (N1−3) = $C/(1+A+B+C)$
Prob (N0) = $1/(1+A+B+C)$
Prob (N+) = 1 − Prob (N0).

**Table IV** Number of node-positive observed (Obs) and predicted (Exp) cases in each decile of probability predicted by the model, in training and test samples

| *Risk deciles* | *Training* *Obs* | *Exp* | *Test* *Obs* | *Exp* |
|---|---|---|---|---|
| 1° | 27 | 24.5 | 7 | 6.6 |
| 2° | 45 | 46.3 | 20 | 19.2 |
| 3° | 31 | 34.1 | 20 | 17.1 |
| 4° | 53 | 54.3 | 22 | 18.8 |
| 5° | 50 | 48.8 | 20 | 16.8 |
| 6° | 52 | 51.3 | 16 | 15.6 |
| 7° | 63 | 62.4 | 19 | 22.0 |
| 8° | 60 | 60.8 | 15 | 13.9 |
| 9° | 72 | 71.5 | 23 | 24.6 |
| 10° | 60 | 60.1 | 28 | 27.2 |
| Hosmer−Lemeshow test | 1.26 ($P$>0.95) | | 8.91 ($P$>0.5) | |

**Table V** Classification accuracy of the predicted probability of nodal metastases

| *Predicted category* | Observed category N0 (n = *471*) | N 1−3 (n = *369*) | N 4−9 (n = *230*) | N10+ (n = *218*) |
|---|---|---|---|---|
| N0 | 84.5 | 56.4 | 38.7 | 32.5 |
| N1−3 | 11.7 | 31.7 | 33.0 | 25.7 |
| N4−9 | 2.3 | 5.4 | 12.6 | 9.2 |
| N10+ | 1.5 | 6.5 | 15.7 | 32.6 |
| Total | 100.0 | 100.0 | 100.0 | 100.0 |

Only column percentages are shown.

similar to the behaviour of the true classes of nodal involvement (Figure 1). Furthermore, survival of misclassified node-negative cases (i.e observed node-negative predicted node-positive) is significantly worse ($P$=0.04) than that of correctly classified node-negative patients (i.e. observed and predicted node-negative) but not significantly different ($P$=0.63) from the survival of true node-positive patients with 1−3 metastatic nodes (Figure 2).

### Discussion

Despite the progressive tendency towards less extensive locoregional treatment for breast cancer, ALND is still routinely performed during breast cancer surgery. The rationale for this surgical procedure, however, has radically changed. In fact, several clinical trials that aimed at defining the curative role of ALND failed to show any significant difference in clinical outcome between patients with or without locoregional treatment to the axilla (Cancer Research Campaign Working Party, 1980; Fisher *et al.*, 1985). Although these results cannot be considered conclusive, it is generally accepted that ALND is of questionable therapeutic value, at least for clinically node-negative breast cancer patients (Lin *et al.*, 1993); nevertheless it is performed for staging purposes and, possibly, to improve the locoregional control of the disease. The latter aspect may be questioned. In the NSABP study (Fisher *et al.*, 1985) clinically node-negative patients randomised to radical mastectomy were found to be pathologically node-positive in 39% of cases; similar figures have been reported by other authors. Yet in the same trial only 17.8% of patients

**Figure 1** Survival curves according to observed (**a**) and predicted (**b**) nodal classes.

**Figure 2** Survival curves of correctly classified node-negative (N–/pN–), misclassified node-negative (N–/pN+) and node-positive with 1–3 metastases (N+(1–3)) subgroups of patients. (N–/pN+ *vs* N–/pN– $P$=0.04; N–/pN+ *vs* N+ (1–3) $P$=0.64).

randomised to simple mastectomy developed axillary relapse requiring delayed dissection. These data strongly suggest that progression of occult axillary metastases occurs at a rate substantially lower than could be expected. Moreover, delayed axillary treatment does not affect the final outcome of the patients experiencing such a progression. Owing to the wide diffusion of breast cancer screening programmes, however, the rate of patients presenting with occult axillary metastases is expected to decrease progressively (Tabar *et al.*, 1985; Tabar *et al.*, 1992; Ahlgren *et al.*, 1994). Since only a small percentage of those metastases would progress to become clinically evident, it seems that the number of patients potentially benefiting from a prophylactic ALND, at least in terms of disease control, will become insignificant.

Also the role of ALND as a staging procedure has been questioned recently (Deckers, 1991; Cady, 1995). In fact, ALND is an important cause of reduced quality of life in women treated for breast cancer and recurrence free. Restricted shoulder mobility, arm oedema, weakness, sensory disturbance and problems with activities of daily life are common (Mazeron *et al.*, 1985; Kissin *et al.*, 1986). In addition, ALND prolongs surgical operating time and hospitalisation. Consequently, attempts have been made to replace the information derived from axillary lymph node examination with surrogate information obtainable at a lower morbidity cost (Rose *et al.*, 1983; Todd *et al.*, 1987; Van Dongen, 1987; Shek and Godolphin, 1988; Tjandra *et al.*, 1989; Toma *et al.*, 1991; Axelsson *et al.*, 1992; Kiricuta and Tausch, 1992).

In this study we investigated the association between some morphological characteristics of the primary tumour and of surrounding tissue and the degree of axillary node involvement, and we attempted to derive a predictive index of the latter from a panel of the former. Although we could not find a perfect predictor of nodal involvement, we demonstrate that knowledge of a few characteristics of the primary tumour provides prognostic information equivalent to nodal status with the same discriminatory ability. Other investigators are pursuing similar goals by using both pathological and biological characteristics (Chadha *et al.*, 1994; Menard *et al.*, 1994). In our study only pathological variables were investigated, because they were retrospectively available in a large file and the results could be generalised to most clinical centres.

Seventeen histological features were studied in a large number of subjects and an integrated multivariate statistical strategy–exploratory and modelling approach–was used to adjust for strong relationships among variables and to optimise the quality of the variable selection. Rather than the simple use of negative/positive alternatives, four levels of axillary node metastases were considered as a response variable, according to the usual classification (N0, N1–3, N4–9, N$\geqslant$10); these four classes identify subgroups of patients with clearly different prognoses and they are often used as a stratification criterion in prospective randomised clinical trials. Even though a possible misclassification of nodal classes may be hypothesised in relation to the ability of the surgeon, degree of nodal involvement was considered in this study as a gold standard with which to compare the quality of the classification process. The only constraint was a minimum number of dissected nodes equal to 6, aiming to reduce the risk of nodal involvement underestimation.

This cutoff might also explain the apparently high rate of node positivity in our series (64%). Similar rates, indeed, have been reported by other authors when the minimum number of lymph nodes examined approximates to ten (Mathiensen *et al.*, 1990; Axelsson *et al.*, 1992). They have also shown that these rates tend to remain stable for higher numbers of examined nodes, suggesting that they probably reflect the true rate of node positivity at the time of primary surgery in these old series of patients.

Evaluation of predictive accuracy of the final model consisted of reliability of prediction and classification accuracy: the former being the consistency of observed proportions of nodal involvement in groups (deciles) of risk with the group's proportions predicted by the model; the latter being the ability of the model to discriminate patients with different degrees of nodal involvement.

Overall, two of our results are clinically relevant: firstly, the observation that the clinical outcome of predicted categories is very similar to the outcome of the observed ones (Figure 1) indicates that the predictive index is as accurate as nodal status; secondly, because the outcome of node-negative patients who are predicted to be node-positive is closer to that of patients with 1 – 3 metastatic nodes than to that of node-negative patients (Figure 2), it is possible, by using the index, to select a subgroup of node-negative patients with unfavourable prognosis. These results imply that ALND as a prognostic procedure could be avoided.

Our index can be improved upon for optimal lymph node involvement prediction: firstly, by using information on clinical nodal status, that was lacking in our retrospective series; secondly, by adding biological variables that have already been shown to be useful for this kind of prediction. For instance Ravdin *et al.* (1994) have recently demonstrated on a very large series of breast cancer patients that some commonly used biological variables (progesterone receptors and S-phase fraction) can significantly contribute to the prediction of nodal involvement. Other factors that could be potentially useful in this task are overexpression/amplification of the oncogene c-*erb*B-2 (Berger *et al.*, 1988; Borg *et al*, 1991), low levels of expression of the *nm23* gene (Hennessy *et al.*, 1991; Royds *et al.*, 1993), alterations of tumour cell surface glycosylation (Alam *et al.*, 1990; Brooks and Leathem, 1991) and tumour neoangiogenesis (Weidner *et al.*, 1991, 1992; Bosari *et al.*, 1992; Horak *et al.*, 1992).

Finally, use of new data integration techniques, like neural networks (NNs) (Rumelhart *et al.*, 1986; White, 1989), could be helpful in producing models to predict the degree of lymph node involvement. Neural networks have recently been applied to the solution of several problems in the biomedical field. Ravdin and coworkers (Ravdin and Clark, 1992; Ravdin *et al.*, 1992; Ravdin *et al.*, 1993) and De Laurentiis and Ravdin (1994*a*,*b*) have shown the ability of NNs to yield models for predictions for oncological patients. It has also been shown that NNs can improve upon traditional statistical models when the data to be analysed are complex and the outcome variable depends on complicated and unexpected interactions among predictive variables (Clark *et al.*, 1994; De Laurentiis and Ravdin 1994*b*). Preliminary but promising results of NN-based predictive models for nodal involvement have already been proposed (De Laurentiis *et al.*, 1994).

In conclusion, pathological features of the primary tumour can be used to produce a predictive index of axillary lymph node metastasis. This index is as accurate as nodal status in predicting OAS for breast cancer patients and it might avoid the need for ALND if this procedure is performed for staging purposes only. However, the classification accuracy of the model must be improved so as to obtain optimal prediction of the degree of lymph node involvement. At present, the index may be useful in the management of patients in whom axillary dissection has not been performed. It may provide additional information to restricted or inadequate lymph node sampling and it is an indicator of high risk of death in node-negative patients.

**Acknowledgements**

Dr De Laurentiis is recipient of an AIRC (Italian Association for Cancer Research) fellowship; the work was partly funded by a MURST (Ministry of University and Scientific Research of Italy) 60% grant.

### References

AHLGREN J, STAL O, WESTMAN G AND ARNESSON L. (1994). Prediction of axillary lymph node metastases in a screened breast cancer population. *Acta Oncologica*, **33**, 603 – 608.

ALAM SM, WHITFORD P, CUSHLEY W, GEORGE WD AND CAMPBELL AM. (1990). Flow cytometric analysis of cell surface carbohydrates in metastatic human breast cancer. *Br. J. Cancer*, **62**, 238 – 242.

ALDRICH JH AND NELSON FD. (1984). Linear probability logit and probit models. pp. 7 – 45. Sage pubns: Beverly Hills and London.

AXELSSON CK, MOURIDSEN HT AND ZEDELER K. (1992). Axillary dissection of level i and ii lymph nodes is important in breast cancer classification. *Eur. J. Cancer*, **28A**, 1415 – 1418.

AZZOPARDI JG, CHEPICK OF, HARTMANN WH, JAFAREY NA, LLOMBART-BOSCH A, OZZELLO L, RILKE F, SASANO N, SOBIN LH, SOMMERS SC, STALSBERG H, SUGAR J AND WILLIAMS AO. (1982). The World Health Organization histological typing of breast tumors – second edition. *Am. J. Clin. Pathol.*, **78**, 806 – 816.

BERGER MS, LOCHER GW, SAURER S. GULLICK WJ, WATERFIELD MD, GRONER B AND HYNES NE. (1988). Correlation of c-*erb*B-2 gene amplification and protein expression in human breast carcinoma with nodal status and nuclear grading. *Cancer Res.*, **48**, 1238 – 1243.

BLOOM HJG AND RICHARDSON WW. (1957). Histological grading and prognosis in breast cancer: a study of 1409 cases of which 539 have been followed for 15 years. *Br. J. Cancer*, **11**, 359 – 377.

BORG A, BALDETORP B, FERNO M, KILLANDER D, OLSSON H AND SIGURDSSON H. (1991). *Erb*B-2 amplification in breast cancer with a high rate of proliferation. *Oncogene*, **6**, 137 – 143.

BOSARI S, LEE AK, DELELLIS RA, WILEY BD, HEATLEY GJ AND SILVERMAN ML. (1992). Microvessel quantitation and prognosis in invasive breast carcinoma. *Hum. Pathol.*, **23**, 755 – 761.

BOUROCHE JM AND SAPORTA G. (1980). L'analyse des donnqes. PUF: Paris.

BROOKS SA AND LEATHEM AJ. (1991). Prediction of lymph node involvement in breast cancer by detection of altered glycosylation in the primary tumour. *Lancet*, **338**, 71 – 74.

CABANES PA, SALMON RJ, VILCOQ JR, DURAND JC, FOURQUET A, GAUTIER C AND ASSELAIN B. (1992). Value of axillary dissection in addition to lumpectomy and radiotherapy in early breast cancer. *Lancet*, **339**, 1245 – 1248.

CADY B. (1995). The need to reexamine axillary lymph node dissection in invasive breast cancer (editorial). *Cancer*, **73**, 505 – 508.

CANCER RESEARCH CAMPAIGN WORKING PARTY. (1980). Cancer Research Campaign (King's/Cambridge) trial for early breast cancer: a detailed update at the tenth year. *Lancet*, 55 – 60.

CHADHA M, CHABON AB, FRIEDMANN P AND VIKRAM B. (1994). Predictors of axillary lymph node metastases in patients with t1 breast cancer: a multivariate analysis. *Cancer*, **73**, 350 – 353.

CLARK GM, HILSENBECK SG, RAVDIN PM, DE LAURENTIIS M AND OSBORNE CK. (1994). Prognostic factors: Rationale and methods of analysis and integration. *Breast Cancer Res. Treat.*, **32**, 105 – 112.

COX DR AND SNELL EJ. (1989). Analysis of binary data. Chapman and Hall: London.

DE LAURENTIIS M AND RAVDIN PM. (1994*a*). A technique for using neural network analysis to perform survival analysis of censored data. *Cancer Lett.*, **77**, 127 – 138.

DE LAURENTIIS M AND RAVDIN PM. (1994*b*). Survival analysis of censored data: neural network detection of complex interaction between variables. *Breast Cancer Res. Treat.*, **32**, 113 – 118.

DE LAURENTIIS M, DE PLACIDO S, GALLO C, PERRONE F, CARLOMAGNO C, BIANCO AR, CLARK GM AND RAVDIN PM. (1994). A predictive model of axillary lymph node involvement in breast cancer patients. *Ann. Oncol.*, **5**, 15.

DECKERS P. (1991). Axillary dissection in breast cancer: when, why, how much, and for how long? Another operation soon to be extinct? *J. Surg. Oncol.*, **48,** 217–219.

FENTIMAN IS. (1993). Axillary surgery in breast cancer: what debate? *Eur. J. Cancer*, **29A,** 923.

FENTIMAN IS AND CHETTY U. (1992). Axillary surgery in breast cancer - is there still a debate? *Eur. J. Cancer*, **28A,** 1013–1014.

FENTIMAN IS AND MANSEL RE. (1991). The axilla: not a no-go zone. *Lancet*, **337,** 221–223.

FENTIMAN IS, HAYWARD JL, RODGER A, SACKS NPM, BAUM M, BARR LC, OZA AM, RAWLINGS G, TANNOCK IF, WHITAKER SJ, WILSON CBJH, HUDDART RA, YARNOLD JR, CABANES PA, SALMON RJ, AKTAN AO, YEGEN C, YALIN R AND BOZKURT S. (1992). Axillary node dissection in breast cancer. *Lancet*, **340,** 245–246.

FISHER B. (1992). The evolution of paradigms for the management of breast cancer: a personal perspective. *Cancer Res.*, **52,** 2371–2383.

FISHER B, REDMOND C, FISHER ER, BAUER M, WOLMARK N, WICKERHAM DL, DEUTSCH M, MONTAGUE E AND MARGOLESE RA1 R. (1985). Ten-year results of a randomized clinical trial comparing radical mastectomy and total mastectomy with or without radiation. *N. Engl. J. Med.*, **312,** 674–681.

GREENACRE M. (1992). Correspondence analysis in medical research. *Stat. Methods Med. Res.*, **1,** 97–117.

HENNESSY C, HENRY JA, MAY FE, WESTLEY BR, ANGUS B AND LENNARD TW. (1991). Expression of the antimetastatic gene nm23 in human breast cancer: an association with good prognosis. *J. Natl Cancer Inst.*, **83,** 281–285.

HLADIUK M, HUCHCROFT S, TEMPLE W AND SCHNURR BE. (1992). Arm function after axillary dissection for breast cancer: a pilot study to provide parameter estimates. *J. Surg. Oncol.*, **50,** 47–52.

HORAK ER, LEEK R, KLENK N, LEJEUNE S, SMITH K, STUART N, GREENALL M, STEPNIEWSKA K AND HARRIS AL. (1992). Angiogenesis, assessed by platelet/endothelial cell adhesion molecule antibodies, as indicator of node metastases and survival in breast cancer. *Lancet*, **340,** 1120–1124.

KAPLAN EL AND MEIER P. (1958). Non parametric estimation from incomplete observation. *J. Am. Stat. Assoc.*, **53,** 457–481.

KIRICUTA CI AND TAUSCH J. (1992). A mathematical model of axillary lymph node involvement based on 1446 complete axillary dissections in patients with breast carcinoma. *Cancer*, **69,** 2496–2501.

KISSIN MW, QUERCI DELLA ROVERE G, EASTON G AND WESTBURY G. (1986). Risk of lymphedema following the treatment of breast cancer. *Br. J. Surg.*, **73,** 580–585.

LEMESHOW S AND HOSMER DW JR. (1982). A review of goodness of fit statistics for use in the development of logistic regression models. *Am. J. Epidemiol.*, **115,** 92–106.

LIN PP, ALLISON DC, WAINSTOCK J, MILLER KD, DOOLEY WC, FRIEDMAN N AND BAKER RR. (1993). Impact of axillary lymph node dissection on the therapy of breast cancer patients. *J. Clin. Oncol.*, **11,** 1536–1544.

MANTEL N. (1966). Evaluation of survival data and two new rank order statistics arising in its consideration. *Cancer Chem. Rep.*, **50,** 163–170.

MARGOLESE RG. (1993). Axillary surgery in breast cancer–there still is a debate. *Eur. J. Cancer*, **29A,** 801.

MATHIENSEN O, CARL J, BONDERUP O AND PANDURO J. (1990). Axillary sampling and the risk of erroneous staging of breast cancer. *Acta Oncol.*, **29,** 721–725.

MAZERON JJ, OTMEZGUINE Y, HUART J AND PIERQUIN B. (1985). Conservative treatment of breast cancer: results of management of axillary lymph node area in 3353 patients. *Lancet*, **2,** 1387.

MENARD S, BUFALINO R, RILKE F, CASCINELLI N, VERONESI U AND COLNAGHI MI. (1994). Prognosis based on primary breast carcinoma instead of pathological nodal status. *Br. J. Cancer*, **70,** 709–712.

RAVDIN PM AND CLARK GM. (1992). A practical application of neural network analysis for predicting outcome of individual breast cancer patients. *Breast Cancer Res. Treat.*, **22,** 285–293.

RAVDIN PM, CLARK GM, HILSENBECK SG, OWENS MA, VENDELY P, PANDIAN MR AND MCGUIRE WL. (1992). A demonstration that breast cancer recurrence can be predicted by neural network analysis. *Breast Cancer Res. Treat.*, **21,** 47–53.

RAVDIN PM, CLARK GM, HOUGH JJ, OWENS MA AND MCGUIRE WL. (1993). Neural network analysis of DNA flow cytometry histograms. *Cytometry*, **14,** 74–80.

RADVIN PM, DE LAURENTIIS M, VENDELY T AND CLARK GM. (1994). Prediction of axillary lymph node status in breast cancer patients by use of prognostic indicators. *J. Natl Cancer Inst.*, **86,** 1771–1775.

ROBINSON DS, SENOFSKY GM AND KETCHAM AS. (1992). Role and extent of lymphadenectomy for early breast cancer. *Semin. Surg. Oncol.*, **8,** 78–82.

ROSE CM, BOTNICK LE, WEINSTEIN M, HARRIS JR, KOUFMAN C, SILEN W AND HELLMAN S. (1983). Axillary sampling in the definitive treatment of breast cancer by radiation therapy and lumpectomy. *Int. J. Radiat. Oncol. Biol. Phys.*, **9,** 339–344.

ROYDS JA, STEPHENSON TJ, REES RC, SHORTHOUSE AJ AND SILCOCKS PB. (1993). Nm23 protein expression in ductal *in situ* and invasive human breast carcinoma. *J. Natl Cancer Inst.*, **85,** 727–731.

RUMELHART DE, HINTON GE AND WILLIAMS RJ. (1986). Learning representation by back propagating errors. *Nature*, **323,** 533–536.

SHEK LLM AND GODOLPHIN W. (1988). Model for breast cancer survival: relative prognostic roles of axillary nodal status, TNM stage, estrogen receptor concentration and tumor necrosis. *Cancer Res.*, **48,** 5565–5569.

TABAR L, FAGERBERG CJG AND GAD A. (1985). Reduction in mortality from breast cancer after mass screening with mammography: randomized trial from the Breast Cancer Screening Working Group of the Swedish National Board of Health and Welfare. *Lancet*, **1,** 829–832.

TABAR L, FAGERBERG G, DAY N, DUFFY S AND KITCHIN R. (1992). Breast cancer treatment and natural history: new insights from results of screening. *Lancet*, **339,** 412–414.

TJANDRA JJ, RUSSEL IS, COLLINS JP, ANDREW JT, LICHTENSTEIN M, BINNS D AND MCKENZIE IF. (1989). Immuno lymphoscintigraphy for the detection of lymph node metastases from breast cancer. *Cancer Res.*, **49,** 1600–1608.

TODD JH, DOWLE C, WILLIAMS M, ELSTON CW, ELLIS IO, HINTON CP, BLAMEY RW AND HAYBITTLE JL. (1987). Confirmation of a prognostic index in primary breast cancer. *Br. J. Cancer*, **56,** 489–492.

TOMA S, LEONESSA F, ROMANINI A, BADELLINO F, BONASSI S, NICOLO G AND ROSSO R. (1991). Predictive value of some clinical and pathological parameters on upper level axillary lymph node involvement in breast cancer. *Anticancer Res.*, **11,** 1439–1444.

VAN DONGEN JA. (1987). Subclavicular biopsy as a guideline for the treatment of breast cancer. *World J. Surg.*, **1,** 306–308.

WEIDNER N, SEMPLE JP, WELCH WR AND FOLKMAN J. (1991). Tumor angiogenesis and metastasis–correlation in invasive breast carcinoma. *N. Engl. J. Med.*, **324,** 1–8.

WEIDNER N, FOLKMAN J, POZZA F, BEVILACQUA P, ALLRED EN, MOORE DH, MELI S AND GASPARINI G. (1992). Tumor angiogenesis: a new significant and independent prognostic indicator in early-stage breast carcinoma. *J. Natl Cancer Inst.*, **84,** 1875–1887.

WHITE H. (1989). Learning in artificial neural networks: a statistical approach. *Neural Comp.*, **1,** 425–464.